# Guideline on current good radiopharmacy practice (cGRPP) for the small-scale preparation of radiopharmaceuticals

**DOI:** 10.1186/s41181-021-00123-2

**Published:** 2021-02-12

**Authors:** Nic Gillings, Olaug Hjelstuen, Jim Ballinger, Martin Behe, Clemens Decristoforo, Philip Elsinga, Valentina Ferrari, Petra Kolenc Peitl, Jacek Koziorowski, Peter Laverman, Thomas L. Mindt, Oliver Neels, Meltem Ocak, Marianne Patt, Sergio Todde

**Affiliations:** 1 Department of Clinical Physiology, Nuclear Medicine and PET, Copenhagen University Hospital Rigshospitalet, Blegdamsvej 9, DK-2100 Copenhagen, Denmark; 2 GE Healthcare, Pharmaceutical Diagnostics, Oslo, Norway; 3 King’s College London, Division of Imaging Sciences and Biomedical Engineering, London, UK; 4 Center for Radiopharmaceutical Sciences, Paul Scherrer Institute, Villigen, Switzerland; 5 Department of Nuclear Medicine, Medical University Innsbruck, Innsbruck, Austria; 6 Department of Nuclear Medicine and Molecular Imaging, University Medical Center Groningen, Groningen, The Netherlands; 7 MSD Animal Health, Milton Keynes, UK; 8 University Medical Centre Ljubljana, Ljubljana, Slovenia; 9 Independant Consultant, Norrköping, Sweden; 10 Radboud University Medical Center Nijmegen, Nijmegen, The Netherlands; 11 Ludwig Boltzmann Institute Applied Diagnostics, Vienna, Austria; 12 Institute for Radiopharmaceutical Cancer Research, Helmholtz-Zentrum Dresden-Rossendorf, Dresden, Germany; 13 Department of Pharmaceutical Technology, Istanbul University, Istanbul, Turkey; 14 Department for Nuclear Medicine, University Hospital Leipzig, Leipzig, Germany; 15 Tecnomed Foundation, University of Milano – Bicocca, Milan, Italy

**Keywords:** cGRPP, GMP, Radiopharmaceuticals, Radiopharmacy

## Abstract

This guideline on current good radiopharmacy practice (cGRPP) for small-scale preparation of radiopharmaceuticals represents the view of the Radiopharmacy Committee of the European Association of Nuclear Medicine (EANM). The guideline is laid out in the format of the EU Good Manufacturing Practice (GMP) guidelines as defined in EudraLex volume 4. It is intended for non-commercial sites such as hospital radiopharmacies, nuclear medicine departments, research PET centres and in general any healthcare establishments. In the first section, general aspects which are applicable to all levels of operations are discussed. The second section discusses the preparation of small-scale radiopharmaceuticals (SSRP) using licensed generators and kits. Finally, the third section goes into the more complex preparation of SSRP from non-licensed starting materials, often requiring a purification step and sterile filtration. The intention is that the guideline will assist radiopharmacies in the preparation of diagnostic and therapeutic SSRP’s safe for human administration.

## Preamble

The European Association of Nuclear Medicine (EANM) is a professional non-profit medical association that facilitates communication worldwide among individuals pursuing clinical and research excellence in nuclear medicine. The EANM was founded in 1985.

This revised guideline has been written by members of the EANM Radiopharmacy Committee and is intended to assist professionals involved in the preparation and quality control of radiopharmaceuticals.

## Introduction

The preparation and use of radiopharmaceuticals in the European Union are regulated by directives, regulations and rules that have been adopted by member states. The rate and extent of adoption and interpretation of directives varies among countries and each member state may introduce changes, provided the general scope and limits of each directive are maintained. Specific articles have been put in place concerning radiopharmaceuticals that are to receive marketing authorization or are prepared starting from licensed products (radionuclide generators, kits and radionuclide precursors). However, radiopharmaceuticals may also be prepared outside the marketing authorisation track or used outside the indications for which they have been registered. Small-scale preparations at non-commercial sites thereby represent an important segment.

The Guidelines on Good Radiopharmacy Practice (GRPP) issued by the Radiopharmacy Committee of the EANM (EANM Guidelines on Current good radiopharmacy practice (cGRPP) in the preparation of radiopharmaceuticals, 2007) were a useful reference to set quality standards for the small-scale preparation of radiopharmaceuticals and their non-radioactive labelling precursors. Further guidance specific to PET radiopharmaceuticals was published subsequently (Elsinga et al., 2010). However, there have been developments in the intervening years, particularly on regulatory aspects and other EANM guidelines, which made it timely to issue a revised guideline. The last two decades have seen the impact of the implementation of the European Clinical Trials Directive (EU Commission, 2001) with its requirement for GMP in the preparation of radiopharmaceuticals for use in clinical trials. The new Clinical Trials Regulation (EU Regulation, 2014), to be implemented in the near future, for the first time recognises the special characteristics of radiopharmaceuticals and relaxes some of the GMP requirements for investigational medicinal products (EU Commission Delegated Regulation (EU), 2017) in the case of preparation of diagnostic radiopharmaceuticals in hospital settings. This revision also takes into account recent documents related to Good Radiopharmacy Practices such as the PIC/S guide to good practices for the preparation of medicinal products in healthcare establishments, with Annex 3 describing radiopharmaceuticals (PIC/S Guide to good practices for the preparation of medicinal products in healthcare establishments, 2014) and relevant chapters in the European Pharmacopoeia (European Pharmacopoeia, 2016; European Pharmacopoeia, 2016). Additionally, specific EANM guidelines related to GRPP have been published or are in preparation, such as guidelines on automated systems (Aerts et al., 2014), process and method validation (Todde et al., 2017; Gillings et al., 2020) and risk assessment (EANM Guideline on “Quality Risk Management guidelines applied to radiopharmaceuticals”, 2021), and are taken into account in this revision.

The general format of the revised guideline follows the structure of the European Union Good Manufacturing Practice (EU GMP) guidelines as laid out in the EudraLex volume 4 (EU GMP, n.d.). The guideline applies to any in-house radiopharmaceuticals not intended for commercial use and is divided into three parts. In Part I general issues which apply to all types of radiopharmaceutical preparations for diagnostic and therapeutic use are addressed. Part II covers small-scale radiopharmaceutical preparations (SSRP) from licensed generators or radionuclide precursors and kit preparations. Part III addresses the preparation of SSRP from non-licensed starting materials. This guideline supersedes all versions of cGRPP published previously by the EANM Radiopharmacy Committee (EANM Guidelines on Current good radiopharmacy practice (cGRPP) in the preparation of radiopharmaceuticals, 2007; Elsinga et al., 2010).

## Part 1 general guidelines

### Pharmaceutical quality assurance system

To consistently and reliably prepare medicinal products of high quality, there should be a comprehensively designed and correctly implemented pharmaceutical quality assurance (QA) system. The quality assurance system must include a systematic process for risk management (ICH Guideline Q9 on quality risk management, 2014). QA represents the sum of all organised arrangements made with the objective of ensuring that medicinal products are of the quality required for their intended purpose. More details can be found in (PIC/S Guide to good practices for the preparation of medicinal products in healthcare establishments, 2014; EU GMP, n.d.).

The extent of the QA system will depend on the scale and complexity of the operations carried out, and will thus differ between Part II and III. However, each system must address at an appropriate level all the topics below. A small scale radiopharmacy must have a responsible person for radiopharmaceuticals (RPR) with the following responsibilities:

- To establish procedures for the examination and evaluation of incoming materials and ensure that each lot of incoming material is examined and evaluated against specifications before use.
- To review the preparation batch records and laboratory control records for accuracy, completeness, and conformance to established specifications before authorizing the final release or rejection of a batch or lot of SSRP.
- To approve procedures, specifications, processes, and methods including related standard operating procedures (SOPs).
- To ensure that all personnel are appropriately trained and qualified.
- To investigate errors and ensure that appropriate corrective actions are taken to prevent their reoccurrence.
- To ensure that SSRPs have adequately defined identity, strength, quality and purity.

Additionally, the person responsible for the QA system at the radiopharmacy should have the following responsibilities:

- To manage the general QA system.
- To verify that the documentation is correctly written and administered
- To conduct periodic audits to monitor compliance with established procedures and practices.
- To monitor, in cooperation with the other staff members, the general management of the activities performed in the small-scale radiopharmacy (e.g. personnel training, radioactive waste management, etc.)

The RPR will most often also be responsible for the QA system. For more efficient management, some QA tasks may be delegated to other staff members or outsourced to help the RPR to oversee and implement the areas mentioned above.

#### Deviations, changes, OOS and CAPA

Written procedures for dealing with deviations should be in place. Deviations from the production protocols should be documented both to identify trends and to guarantee that corrective and/or preventative actions will take place. A change management system should be in place to deal with all changes that may affect the quality of the SSRP. This includes changes in preparation methods as well as in QC, equipment, software, manufacturing and suppliers. Systems for handling OOS (out of specification) results and corrective and preventative actions (CAPA) should be in place.

### Personnel

There must be a sufficient number of personnel with the necessary education and appropriate training and experience. The roles in the organization must be described, for example by creating an organizational chart.

Small-scale radiopharmacies must maintain an up-to-date file including curriculum vitae (CV), job description and relevant certificates (training, education, authorizations etc.), for each staff member.

Staffing levels should be appropriate for the activities of the radiopharmacy and there should ideally be a minimum of two, one of whom is the RPR with appropriate credentials. The second person must be well trained in cGRPP procedures. Where only one person is directly involved in preparation of SSRPs, all procedures including preparation, quality control (QC) and release of the product may have to be done by this person (the RPR). In this case rigorous SOPs must be in place and should be strictly adhered to. A second suitably qualified and trained person should review all results to confirm their compliance. This individual could be anyone trained to review appropriate results, understand their meaning and confirm their correctness.

All personnel (including those concerned with cleaning and maintenance) employed in areas where radioactive products are handled (exposed workers) must categorised according to article 40 and monitored according to article 41 of the European Council Basic Safety and Standards Directive (as implemented in national law) (European Council Basic Safety and Standards Directive, 2013) and should receive additional training specific to this class of products. In particular, detailed information and appropriate training on radiation legislation, protection, decontamination, handling and storage of radioactive materials should be given.

Training must be provided for all staff working in a radiopharmacy. This includes:

- Working practices in the radiopharmacy including aseptic techniques and radiation protection
- Radiopharmaceutical preparation and preparation of individual doses
- Quality control and analytical techniques
- Release procedures
- Documentation
- Hygiene, pharmaceutical microbiology and microbiological monitoring
- Calibration and qualification of equipment
- Cleaning
- Transport of radioactive material (IAEA Safety Standards, 2018)

The level of training required will depend on the intended task(s). A description of the training and records of completion and requalification must be kept. The responsibilities should be outlined in job descriptions and/or authorizations, considering clear distinctions between research activities and preparations for clinical use.

A detailed discussion on the requirements for a Medical Physics Expert (MPE) and/or Radiation Protection Expert (RPE) is covered by national and European regulations and is beyond the scope of this guideline.

### Premises and equipment

Facilities should be adequate to assure the orderly handling of materials and equipment, the prevention of mix-ups and the prevention of contamination of equipment or product by substances, personnel or environmental conditions that may have an adverse effect on product quality.

Radioactive products must be stored, processed, packaged and controlled in dedicated facilities. Only the necessary equipment should be located there. Access to controlled areas should be via a gowning area and should be restricted to authorised personnel. Visitors, cleaning staff and technical staff should follow appropriate rules for access, which should be described in written instructions. After maintenance and repairs, premises must be cleaned and decontaminated appropriately.

Appropriate measures should be taken to avoid the spread of radioactivity from controlled areas and to protect them from particulate and microbial contamination. Measures to monitor key environmental parameters (e.g. temperature, humidity, particle levels) should be applied and the frequency of measurement/assessment should be defined. Appropriate equipment should be in place for the detection and monitoring of personnel for radiation exposure.

Workplaces, as appropriate, must be classified into controlled or supervised areas; signs indicating the type of area, the nature of the sources and their inherent risks shall be displayed (article 36–38 of the European Council Basic Safety and Standards Directive (European Council Basic Safety and Standards Directive, 2013)).

The use of the same area for multiple purposes at the same time should be avoided and there should be physical segregation of operations wherever possible. Different operational areas should be clearly defined and separated, especially regarding the unidirectional flow of starting materials, intermediates and finished products to avoid mix-up and unintended use. For example, preparation (e.g. radiochemical synthesis), QC and laboratory operations (e.g. release testing), and storage of approved components (including containers and closures) should be performed in segregated areas wherever possible or otherwise physically separated.

For labelling of blood cells, a dedicated laminar air flow bench should be used, and preferably a dedicated clean room.

Where both research and clinical preparation activities are carried out at the same location, these should be physically or organisationally separated from each other to avoid research activities impacting on the quality of products for clinical use.

Materials should be clearly identified as “In quarantine”, “Approved” or “Rejected” and kept physically separated where possible.

Aseptic manipulations must take place in a grade A environment. The grade of the surrounding area will depend on the containment system used, the method of preparation, the risk of contamination for the preparation, the shelf-life of the preparation and the number of units prepared during a preparation run. Following a risk assessment, a grade C surrounding area for laminar air flow workbenches (LAFW), or a grade D surrounding area for isolators is generally considered suitable (PIC/S Guide to good practices for the preparation of medicinal products in healthcare establishments, 2014). Examples of aseptic manipulations include assembly of sterile components (syringe, needle, filter and vial) for sterile filtration and containment of the SSRP, elution of a technetium-99 m generator, reconstitution or dilution of a kit, removal of QC test samples, and sterility testing of the finished SSRP.

All surfaces including walls, floors, ceilings, tables and furniture in the radiopharmaceutical preparation and quality control areas must consist of materials easy to clean, disinfect and decontaminate in case of a radioactive spill. Cleaning and sanitising must be performed regularly according to approved procedures, and documented to ensure consistent control of environmental quality. Sinks should be outside the preparation area. Secondary packing, such as cartons and boxes, should not be taken into the preparation area, to minimize ingress of dust and other particulate matter. A technical area for maintenance (technical corridor) could be constructed such that hot cells are approached from a room other than the cleanroom.

Operators should wear designated cleanroom clothing appropriate to the grade of cleanroom and sterile or sanitised gloves when conducting aseptic manipulations. Recommendations for appropriate clothing can be found in Annex 1 of the PIC/S guide (PIC/S Guide to good practices for the preparation of medicinal products in healthcare establishments, 2014). Additional protective garments (e.g. safety glasses, visors and arm coverings) should be worn when necessary and in line with radiation protection requirements. Only people directly involved should be present during the preparation of a SSRP.

Areas and equipment for aseptic manipulation should be regularly monitored with respect to microbiological quality and airborne particles (PIC/S Guide to good practices for the preparation of medicinal products in healthcare establishments, 2014).

There must be appropriate procedures for the sanitisation of materials and equipment being transferred into the aseptic work area. An isolator should have a material air lock with interlocking doors for transfer of material.

Items within a laminar air flow aseptic workstation should be kept to a minimum and should not interrupt the airflow delivered to the critical grade A area.

Equipment must be qualified before initial use (Todde et al., 2017) and undergo regular and recorded preventative maintenance and recalibration. The need for recalibration and requalification should be based on risk assessment (EANM Guideline on “Quality Risk Management guidelines applied to radiopharmaceuticals”, 2021). Details on equipment used for measurement of radioactivity as well as automated synthesis and dispensing modules can be found elsewhere (European Pharmacopoeia, 2016; Aerts et al., 2014).

Other equipment used for preparation of radiopharmaceuticals such as water baths, thermometers, heating plates, etc., must be checked for accuracy and records of these should be kept. Equipment used for verification of critical parameters (e.g. thermometers, flowmeters, particle counters, etc.) related to production and QC instrumentation should be regularly calibrated, based on risk analysis, using suitable traceable calibration standards.

A system of planned preventive maintenance and calibration should be operated to ensure that all facilities and equipment used in the preparation and quality control of radiopharmaceuticals are regularly maintained and calibrated where appropriate. Records and logs should be kept for all equipment irrespective of whether maintenance and calibration are performed in-house or by external contractors.

### Documentation

Good documentation practice is critical to provide written proof that activities have occurred. It applies to everyone who documents activities related to cGRPP. A document management system must be in place to ensure traceability of all procedures. Documentation may be paper-based, electronic, or a combination of the two.

Instructions and Standard Operating Procedures (SOPs) should be written and independently approved for each procedure or operation within the small-scale radiopharmacy. These operations include production, quality assurance and management aspects. SOPs should be reviewed at least every 2–3 years unless they require immediate revisions. There must be version control such that only the currently approved version is accessible.

A specification should be available for each starting material/radiolabelling kit used as well as for radiopharmaceutical products.

Among records to be kept are:

- Purchase and control on arrival of all starting materials, excipients and radionuclides/radionuclide generators.
- Product preparation: batch records, results of quality control tests and release decisions
- Laboratory cleaning and maintenance
- Equipment maintenance, calibration, cleaning, qualification and use
- Training of personnel
- Transport of radioactive materials
- Radioactive contamination monitoring and radioactive waste disposal
- Microbiological and particulate monitoring
- Temperature monitoring, particularly refrigerators and freezers
- Product defects and events of non-conformity to SOPs (deviations)
- Qualification/validation protocols and reports (facilities, equipment, processes and analytical methods)
- Change management

Corrections to paper entries should be dated and signed or initialled, leaving the original entry still legible. Records should be retained for a sufficient period to satisfy national legislative requirements. More information on documentation can be found in the PIC/S guide for healthcare establishments (PIC/S Guide to good practices for the preparation of medicinal products in healthcare establishments, 2014).

### Production

All goods received should be checked against the order for correctness of delivery. In addition, a visual inspection should be carried out prior to acceptance. Records should be kept for materials used for preparation. Products or kits with a marketing authorisation should be used whenever possible.

Production of SSRPs should be organized in such a manner as to prevent cross-contamination of the product. Process validation, in-process controls and monitoring of process parameters and environment are particularly important in cases where it is necessary to take the decision to release or reject a batch or a product before all tests are completed, which is often applicable to SSRPs.

All containers for SSRP preparations (including syringes and shields) must be labelled. Labels should be legible and applied to remain legible and affixed during the established conditions of processing, storage, handling, distribution and use (World Health Organization, 2003).

Due to risk of radiation exposure, it is a common practice to prepare much of the labelling in advance.

Dispensing of patient doses should be individual, and all syringes must be identified by product name and amount of radioactivity at a defined time.

Packaging and shipping containers designed and constructed to protect personnel from radiation and the product during storage, distribution and handling should be used.

Any deviation from approved procedures should be avoided. In the event of deviations, these must be documented and approved in writing by a responsible person. Deviations should be investigated where deemed appropriate.

#### Radiolabelling of blood cells

When preparations from patient material (blood cells or platelets) are performed, strict requirements regarding aseptic handling must be followed. All starting materials should be identified. For any reagent, material or solution specifically intended for human use, it must be documented that its specifications meet the required standards. Materials and reagents certified for human use should be used. More details can be found in the respective guidelines (Roca et al., 2010; de Vries et al., 2010), and local legislation must be considered.

Preparation of labelled cells must be performed separately to prevent cross contamination. Samples from different patients must always be handled separately; two preparations must never occur at the same time. Strict procedures must be in place to prevent mix-ups.

### Quality control

Each laboratory performing quality control testing related to the preparation of SSRPs should have and follow written procedures for the conduct of each test and for the documentation of the results. Complete records of all tests necessary to ensure compliance with established specifications and standards, including examinations and assays should be kept.

Each laboratory should have scientifically sound sampling and testing procedures designed to assure that SSRPs conform to specifications.

Laboratory analytical methods should be suitable for their intended use and should be sufficiently sensitive, specific, accurate and reproducible.

All prepared solutions should be properly labelled to show their identity and composition.

All equipment used to perform the testing should be suitable for its intended purpose and capable of producing valid results. Each laboratory should have and follow written procedures to ensure that equipment is routinely maintained, calibrated and qualified, and that these activities are documented.

Specifications and quality control testing procedures for many of the currently used SSRPs are given in specific monographs in the European Pharmacopoeia (Ph. Eur.) and/or the Summary of Product Characteristics (SmPC) distributed with each licensed product. Analytical methods, specifications and acceptance criteria might also be defined internally, provided that they are scientifically sound, validated, and of a comparable level with those described in Ph. Eur. (Guide for the elaboration of monographs on radiopharmaceutical preparations, 2018).

There should be written procedures detailing all preparation and quality control data that should be considered before the preparation is released for human use (release specifications). A procedure should also describe the actions to be taken by the RPR if OOS results are obtained.

#### Sterility and bacterial endotoxin testing

The purpose of sterility testing is to ensure that the procedures used in the radiopharmacy result in sterile products. For most radiopharmaceuticals, it is not possible to obtain results of certain tests, e.g. sterility test, before the product is released. However, these tests should be performed to monitor the preparation process. Usually samples for sterility testing are stored for sufficient radioactivity decay, and then sent for sterility testing to an external laboratory. Internal sterility testing is only recommended for products with half-lives > 7 h, when the external testing laboratory has no radiation license and if it is performed according to the European Pharmacopoeia.

Bacterial endotoxin testing (BET) is specified separately in Section 2.6 and Section 3.6.

#### Finished radiopharmaceutical controls and acceptance criteria

These processes are specified separately in Section 2.6 and Section 3.6.

### Outsourced activities

Any activity that is outsourced should be appropriately defined, agreed and controlled to avoid misunderstandings which could result in a product or operation of unsatisfactory quality. There must be a written contract between the radiopharmacy and the contractor which clearly establishes the duties and responsibilities of each party.

Examples for which a contract could be required are:

- Microbiological and environmental monitoring, sterility testing
- Air handling plant monitoring and preventative maintenance
- Chemical testing, e.g. mass spectrometry, solvent analysis
- Periodic verification of calibrated reference instrumentation (e.g. thermometers, flowmeters, etc.)
- Qualification of instruments and/or ancillary equipment such as HVAC
- Sterilization of starting materials/equipment
- Cleaning of the facility

A system for evaluation and approval of services providers (contractors) should be in place. Auditing prior to approval may be appropriate.

### Complaints and recalls

Written procedures should be followed for the receipt and handling of complaints regarding SSRPs, including collection of information, root cause analysis and corrective and preventative actions. A documented recall procedure should be in place.

### Self-inspection

The QA unit (e.g. the RPR at small facilities) should monitor compliance with the QA system periodically by performing an internal inspection (self-inspection). Self-inspections should be performed in the form of an audit, for which specific topics may be selected (e.g. personnel, equipment, batch records) allowing recognition of shortcomings. Any failures in the QA system must be documented including appropriate measures to overcome them, and any corrective or preventative measures must be reviewed during the next self-inspection. Self-inspections may also be outsourced to a quality department within the hospital, or to an external consultant. Records of self-inspections must be kept.

## Part 2 guidelines for preparation of radiopharmaceuticals from licensed components (generators, radionuclide precursors, and kits)

### Pharmaceutical quality system

See Section 1.1.

### Personnel

See Section 1.2.

### Premises and equipment

All premises and equipment must be designed and used in accordance with requirements for both aseptic processing and radiation safety, including, for example, shielding of generators and final SSRP’s. Areas for preparation, dispensing, quality control etc. should be segregated as appropriate. The surfaces of lead shielding must be covered to avoid exposure to lead and decrease the amount of lead dust in the environment (i.e. painted or covered with aluminium, plastic foil or stainless steel). Care should be taken in aseptic handling of generators according to the manufacturer’s instructions.

### Documentation

In addition to the records listed in Section 1.4, all preparation and quality control activities should be performed according to approved procedures and documented.

### Production

Specific information about the handling of radionuclide generators, including instructions for generator elution, checking of the elution yield and other tests of generator quality is provided in the package insert supplied with the generator. Similarly, the package insert includes detailed information regarding kit labelling procedures. These instructions should be strictly followed, and a procedure should be in place to check that there have been no changes when a new batch is received. If there is a clinical need but a licensed product is unavailable nationally, it should be confirmed that an imported product is of pharmaceutical grade, considering any national regulatory requirements.

All rubber stoppers, including those on the eluate vials, must be wiped with a sterile disinfecting agent immediately before puncturing with a needle or spike. The disinfecting solution should be allowed to evaporate completely before puncture, as the introduction of this agent may influence kit performance. The elution shield and the shields for vials and syringes must be checked for contamination and cleaned inside and out before use, for example with 70% ethanol or isopropyl alcohol.

New personnel must be qualified by media fills and all personnel requalified at regular intervals to verify aseptic handling techniques.

Products should not be prepared in the same workspace simultaneously to prevent product mix-ups.

To minimize exposure to radiation, proper shielding is needed as well as proper planning of the handling of radioactive products. Generator(s) should always be properly shielded, taking into account the decay properties of parent and daughter radionuclides. Radioactive solutions should be stored in individual, suitably labelled shielding. Tongs or tweezers should whenever possible be used when radioactive solutions are handled, e.g. when measuring activity in a dose calibrator.

Transport of eluates and preparations inside the department must take place within shields, e.g. the elution shield, syringe shield.

### Quality control

The quality control procedures described in the SmPC for generator eluates and kit-based radiopharmaceuticals should be performed, as a minimum for each new batch of kits. Alternative quality control procedures may be used provided they are scientifically sound and validated. Additional quality control tests of the generator eluates which are not defined in the SmPC may be considered, for example:

- Elution efficiency of each eluate
- Parent radionuclide breakthrough on the first eluate from each generator and at regular intervals

Sterility and bacterial endotoxin testing is normally not required for preparation of SSRPs using licensed generators and kits, but a regular monitoring of aseptic techniques (e.g. by media fill) is recommended.

#### Release of radiopharmaceuticals

The responsible person for radiopharmaceuticals (RPR) must ensure that the products have been prepared in accordance with the manufacturer’s instructions and that only preparations with suitable quality are released. This is achieved by appropriate training, documentation, quality control, and supervision of the process and staff involved.

### Outsourced activities

See Section 1.7.

### Complaints and recall

See Section 1.8.

### Self inspection

See Section 1.9.

## Part 3 guidelines for preparation of radiopharmaceuticals from non-licensed components

The following guidelines may also be appropriate when deviating from recommended procedures using licensed components.

### Pharmaceutical quality assurance system

There should be a QA unit that can oversee preparation operations to ensure that a SSRP of sufficient quality is prepared. Depending on the size and organisation structure, if a separate QA unit is not possible, then the RPR should assume this responsibility.

The QA unit must:

- Approve or reject procedures or specifications and any changes to a specification, method, process or procedure that affect the identity, concentration, quality or purity of a SSRP before they are implemented
- Assess the need for requalification and/or revalidation after a change has been made
- Review preparation/QC records to determine whether errors have occurred
- Ensure that errors or failures to meet any specification have been fully investigated and corrective/preventative actions have been implemented

### Personnel

There are several key personnel beside the RPR who must be in place and the inter-relationships among them defined.

There must be a Production Manager whose responsibilities include:

- To ensure that SSRPs are produced and stored according to the appropriate documentation to obtain the required quality
- To approve the instructions relating to production operations and to ensure their strict implementation
- To ensure that production records are evaluated and approved by an authorised person
- To ensure the qualification and maintenance of the premises and production equipment
- To ensure that process validation has been performed
- To ensure that the required initial and continuing training of production personnel is carried out and adapted according to need

There must be a Quality Control Manager whose responsibilities include:

- To approve or reject starting materials, packaging materials, intermediate, bulk and finished products
- To ensure that all necessary testing is carried out and the associated records are evaluated
- To approve specifications, sampling instructions, test methods and other quality control procedures
- To approve and monitor any contract analysts
- To ensure the qualification and maintenance of the QC equipment
- To ensure that analytical methods are validated
- To ensure that the required initial and continuing training of the quality control personnel is carried out and adapted according to need

The Production Manager and the Quality Control Manager should normally not be the same person. However, in small facilities the responsibilities for production and/or QC may be assumed by the RPR. Whether the above functions are separated or not, they must be clearly described in an organisational chart.

### Premises and equipment

All equipment used in production (e.g. cyclotron, synthesis units, or other specialized equipment) should be appropriately located and housed (e.g. with shielding) so that all the working areas during the normal course of production are easily accessible. It is recommended that related working areas are organized and proximally located to promote efficient operation and eliminate the potential for errors in preparation and monitoring operations.

Preparation of SSRPs from non-licensed components is usually performed using automated systems (Aerts et al., 2014), located in suitably shielded hot cells with at least a grade C environment. Minimum requirement for the background grade is D. Sterilisation and dispensing should be performed in a Grade A environment (see above). Alternatively, final sterile filtration may be performed in the same grade C environment if the components, including the sterile filter, have been assembled in an area with grade A air supply.

### Documentation

For general guidelines, see Section 1.4.

Written procedures must specify how each material (components, containers and closures) will be selected and controlled in the radiopharmacy. Procedures should cover the life cycle of a material, from the time of receipt to ultimate consumption. A master batch record serves as a template for all batch records documenting how each batch is produced and controlled; specific SOPs should complement this with detailed descriptions of all processes. The RPR should approve the master batch record, and any changes, before they are implemented.

It is recommended that the master batch record also includes descriptions and specifications of critical steps during preparation of the SSRP, thereby complementing the SOP. Critical steps include the process steps, process conditions or other relevant parameters that are controlled within predetermined criteria, to ensure that the final product meets its specification. Preparation should be described under headings, where applicable, such as cyclotron operation, radiochemical synthesis, purification steps, formulation of the finished product and QC. The SOP for performing a specific step can be referenced.

Batch records should document each significant step in the preparation and quality control (QC) of a SSRP (batch production and QC record). A signed entry including the time should be made in the batch record directly after performing an activity (the entries being in the order the activities were performed). Each completed batch record is reviewed and approved for final release (signature/ initials and date). Requirements for batch production and QC records are summarised in Table 1.

**Table 1:** Requirements for a batch production and QC record

**Production**	
The name, batch number, total radioactivity, volume and expiry time and date of the SSRP	
The name and weight or measure of each active ingredient and component as well as a statement of the total weight or measure of any dosage unit	
Records of any checks and system suitability tests performed	
The batch numbers of all components used (expiry dates may be included)	
A complete list of components and equipment designated by sufficiently specific names or codes	
Names (initials or signatures) of persons performing or checking each significant step in the operation and any investigations conducted	
A statement of practical yield, including the maximum and minimum percentages of practical yield beyond which investigation is required	
Deviations from written procedures	
**Quality Control (QC)**	
A description of the sample received for testing including its source, batch or lot number, date and time the sample was taken	
A description of each method used in the testing of the sample, a record of all calculations performed in connection with each test and a statement of the weight or measure of the sample used for each test, if relevant	
Records of any checks and system suitability tests performed	
A record of relevant data obtained during each test, including graphs, charts, and spectra from laboratory instrumentation, properly identified to show the specific component, in-process material, or SSRP for each lot tested	
A statement of the test results and how the results compare with established acceptance criteria	
The initials or signature of the person performing the test and the date and time of the test	
Deviations from written procedures	
A clear statement of approval or rejection including the time and date of release and the signature of the person designated to release the batch	

### Production

Production and process controls should ensure the consistent preparation of an SSRP that meets the quality standards. They should include monitoring of all measurable parameters such as pressures, temperatures, radioactivity levels, and gas and liquid flow rates at relevant process locations and times. Documentation should include written production and process control procedures (SOPs), master batch records, and validation of the production process and controls.

Each time a batch of a SSRP is produced, a unique batch record must be prepared that includes details of production and quality controls as listed in Table 1.

The process for producing and controlling each SSRP should be validated according to established procedures and approved by the RPR. The EANM Radiopharmacy Committee has issued a guidance on validation and qualification of processes and operations involving small-scale preparation of radiopharmaceuticals (Todde et al., 2017).

#### Microbiological control of aseptic processing and sterilising filtration

Aseptic processing of SSRPs should include microbiological control of relevant components.

The selection of reliable vendors and high-quality materials are effective ways to limit the risk of microbiological contamination. Sterile, single use components are preferable. Components that support microbial growth during storage should be kept under controlled conditions and periodically assessed for microbial growth and/or contamination.

Only personnel trained in aseptic techniques and qualified by media fill should conduct aseptic processing as described in (Todde et al., 2017). Aseptic processing in SSRP preparation using non-licensed components normally consists of but is not limited to: (1) the aseptic assembly of the container/closure system (syringe, needle, filter and vial) and (2) sterile filtration of the SSRP.

An SSRP is non-sterile until it is passed through a sterile filter. Generally, small-scale radiopharmacies use commercially available disposable sterile filters. The filter must be compatible with the product. If sterile filtration is not possible due to the nature of the product, the bioburden of the starting materials and final product should be strictly controlled.

A filter integrity test should be performed immediately after the filtration process and before use of the SSRP, provided that the operation is in compliance with radiation safety requirements. This can be accomplished by performing a pressure-hold test or a bubble-point test to show that the integrity of the filter was not compromised before or during use. Calibrated equipment is available to perform the filter integrity test, or it may be part of the programme of an automated synthesis module.

Aseptic processing of SSRPs should include microbiological control of components, as discussed above. Additionally, the bioburden of the processing system and/or critical steps in the process should be analysed as described in (Aerts et al., 2014). In this context, special attention should be paid to the components described below:

- Transfer lines, valves and HPLC system
  Transfer lines, which are used for synthesis and transfer of solvents or products, are usually made of durable plastic and are suitable for re-use. Appropriate cleaning with water for injection, antimicrobial substances, flushing with a volatile solvent, and drying with an inert gas (e.g. nitrogen, helium, argon) helps control microbial contamination. Organic solvents such as ethanol and acetone are useful as a final rinse and can easily be dried from containers or lines. For SSRPs with a very short half-life ([^15^O] water and [^13^N]ammonia), a long transfer line may be used to deliver multiple batches to a remote area for further processing. It is essential that validated cleaning procedures are established to ensure that these transfer lines are clean and free of microbial and bacterial endotoxin contamination.
- Resin and other columns
  Resin columns are a potential source of microbes and bacterial endotoxins. If available, the purchase of resin material of low microbial grade may limit the bioburden. Material used for preparing resin columns should be suitably processed and rinsed with a large amount (> 10 times the column bed volume) of sterile water for injection to minimize contamination risk. The prepared column should be appropriately flushed. Refrigerated storage is helpful in controlling contamination. It is recommended that wet columns are not stored for a prolonged period. HPLC columns used for purification of SSRPs and outlet lines, although somewhat less susceptible to bacterial contamination should also be monitored for bioburden and disinfected regularly with, for example 70% ethanol.
- Aqueous solutions
  Water for injection should be used whenever possible. Once a container with water for injection has been opened, or when a volume of water has been dispensed, the container should be used within the same day.
- Glassware
  Single use items should be used wherever possible. Re-usable reaction vials from a synthesis module made of glass should be cleaned using validated methods. Cleaning procedures should not affect the quality of the SSRP. It is recommended that glassware is dried and depyrogenised in a dry-heat oven.

### Quality control

Batch records should include all tests necessary to ensure compliance with established specifications and standards, see Table 1.

Test methods should be validated unless included in the appropriate European Pharmacopoeia monograph for the product. Reference materials should be established (where available) for validation and routine testing. Materials, reagents and reference standards used for QC tests should be of appropriate quality. Where necessary, identity verification and other tests should be considered on incoming materials before use in the preparation of SSRP’s.

#### Control of starting materials

The process for procurement and use of materials should include the following elements, where applicable as described below:

- Vendor selection
  For all starting materials and critical components, only qualified vendors should be used. Vendor qualification can be established by an audit, by responses to a QA questionnaire, or simply based on experience with this supplier (e.g. the hospital pharmacy). In any case, vendor qualification should be documented.

- Receipt of starting materials
  Each lot of material should be checked upon receipt to confirm that the order was fulfilled correctly and arrived in good condition. Each lot should be logged and assigned a new identification code number. Sufficient information should be documented to enable the radiopharmacy to have full traceability of each lot. It is recommended that incoming materials are segregated and placed in an appropriately designated area under quarantine and labelled “In quarantine” until released. Specifications and acceptance criteria for starting materials should be defined and supplier certificates of analysis should be checked to ensure that they comply with their specifications. Additionally, for critical starting materials, e.g. in case of reagent sets for cassette automated systems, a test labelling, followed by analytical testing of the radiopharmaceutical can be considered.

- Release of starting materials
  All materials that meet a radiopharmacy’s specifications can be approved and released for use by the Quality Control Manager. Such release should be recorded, and examination, testing data and release should be documented. It may be helpful to have a component logbook to record information such as receipt date, quantity, supplier’s name, lot number, expiry date, results of any testing performed, and person responsible for release. Approved materials can be labelled “approved” with an identifying code number, storage conditions, and expiry date. Materials should be stored under appropriate conditions to prevent degradation or contamination, and in an area designated for approved materials. If a lot is rejected, it should be labelled “rejected”, segregated and properly disposed of. Each of these actions must be documented.

#### Validation of analytical methods

Analytical methods must be validated unless described in the European Pharmacopoeia. The EANM Radiopharmacy Committee has published a guideline on the validation of analytical methods for radiopharmaceuticals (Gillings et al., 2020).

#### Reference standards

Many analyses use reference standards. It is recommended that radiopharmacies establish reference standards identified in the analytical procedure or SOP, or described in the European Pharmacopoeia. If a radiopharmacy establishes its own reference standards, it is recommended that data to confirm the material’s identity and purity should be established and documented. Documentation such as reference spectra or other supporting data to prove the identity and purity of the reference standard may be available from the supplier.

Radioactivity measurements with dose calibrators and tests for radionuclidic purity require calibrated radioactive sources, to be used as reference standards.

#### SSRP stability

As with other drug products, SSRPs are expected to remain stable during shelf life. However, certain SSRPs (e.g. [^18^F]fluorodopa) can undergo autoradiolysis during storage (especially at high radioactivity concentrations). Therefore, appropriate parameters should be evaluated to establish and document the stability of SSRPs under the proposed storage conditions and separate release specifications should be considered. Examples of stability parameters include radiochemical purity (including levels of radiochemical impurities), appearance, pH, stabilizer or preservative effectiveness and chemical purity. It is required that appropriate stability-indicating methods that can distinguish degradation products and impurities are used. Stability testing of SSRPs should be performed at the highest levels of radioactivity. The final product should be studied for a time equal to the shelf life of the SSRP under worst-case conditions. Although stability studies in support of an expiry period would be needed for approval of a SSRP, subsequent changes to the shelf life, product composition or radioactivity concentration should only be made after adequate stability testing procedures, as described above.

#### Controls and release criteria for finished SSRPs

Release criteria should be established for SSRPs including criteria for identity, concentration (chemical and radioactivity) and purity. Each batch of a SSRP should meet its established release criteria before it is released. Any tests which are to be performed post release must be specified following a risk assessment unless defined in the respective monograph of the European Pharmacopoeia (e.g. sterility testing).

SSRPs that have a very short half-life (e.g. [^13^N]ammonia) can be produced in multiple batches on the same day. End product testing of the first and last batch of the day can be conducted, provided that process validation has demonstrated that the product consistently meets the predetermined acceptance criteria. Subsequent batches can then be released throughout the day without the need to perform full quality control testing.

The SSRP may not be released unless:

- Appropriate laboratory testing is completed, and the product meets its specifications
- Associated production and laboratory data and documentation are reviewed
- The product has been prepared according to defined procedures
- Release is authorised by the dated signature of the RPR

In specific cases, modifications to this standard procedure for product release may be appropriate. For example, transportation deadlines may justify distribution before all testing and review are finalised. These tests should be completed prior to release for human administration.

#### Actions if a batch of SSRP does not meet the acceptance criteria

If a batch of SSRP does not meet the acceptance criteria (i.e. an OOS result), the product should be clearly labelled and set aside to avoid mix-ups, until the RPR has been notified and decided upon rejection. An SOP describing the actions to be taken and to investigate the cause(s) must be in place, e.g. in a decision tree. Such an investigation should include, but is not limited to, examination of processes, operations, records, complaints and any other relevant sources of information and must be documented. Further action should be taken to correct any identified problems to prevent reoccurrence of the non-conforming product.

Under limited circumstances an SSRP can be reprocessed if pre-established procedures (set out in production and process controls) are followed and the finished product conforms to specifications before final release. When the option for re-processing is exercised, the event must be documented, and the conditions described in a deviation report. Examples of re-processing might include a second passage through a purification column to remove an impurity, or a second passage through a filter if the original filter failed the integrity test.

#### Sterility and bacterial endotoxin tests for sterile SSRPs

Sterility testing should start as soon as feasible after the completion of the SSRP production. Aseptic techniques should be used for sterility testing and should comply with the standards of the European Pharmacopoeia. If sterility testing is outsourced, the samples should be allowed to decay completely before shipping, unless the external laboratory has a license for handling radioactivity. For longer lived SSRP’s, where testing of radioactive samples is not feasible, alternative strategies may be considered, e.g. mediafill simulation using all materials except the radionuclide precursor.

Although the traditional limulus amoebocyte lysate (LAL) gel-clot test requires approximately 1 h, more rapid tests are now available such as the kinetic chromogenic assay, which only take around 15 min and are often practical to perform before release. Therefore, a bacterial endotoxin test (BET) should ideally be performed before release of the SSRP, in particular when radionuclides are produced on-site by cyclotron (which is not considered part of the GMP system), if reusable equipment is involved, or if there is a resin column purification step.

If the result of a BET exceeds the acceptance limit, or if a sterility test is positive for microbial growth, a complete investigation should be conducted immediately and documented. It is recommended that corrective and preventative actions based on the results of the investigations are implemented promptly.

#### Reference samples

In the case of preparations without a marketing authorisation, retention samples of SSRP’s should be kept for a minimum period of 1 month from the time all testing is completed or minimum 1 month after expiry of the preparation, whichever is the longer. In the case of single vial dispensing, reference samples may not be available; this should be carefully considered in any risk assessment. No retention samples are required for preparations of radiolabelled blood cells.

Where technically possible, the same approach should be applied to reference samples of chemical precursors and starting materials.

### Outsourced activities

See Section 1.7.

### Complaints and recall

See Section 1.8.

### Self-inspection

See Section 1.9.

## Data Availability

Not applicable.

## Abbreviations

API: Active pharmaceutical ingredient
BET: Bacterial endotoxin test
CAPA: Corrective and preventative action
cGRPP: Current good radiopharmacy practice
EANM: European association of nuclear medicine
GMP: Good manufacturing practice
HEPA filter: High efficiency particulate air filter
HPLC: High performance liquid chromatography
LAFW: Laminar air flow workbench
LAL: Limulus amoebocyte lysate
OOS: Out of specification
Ph. Eur.: European pharmacopoeia
QA: Quality assurance
QC: Quality control
RPR: Responsible person for radiopharmaceuticals
SOP: Standard operating procedure
SmPC: Summary of product characteristics
SSRP: Small-scale radiopharmaceutical
